# Facile Conversion and Optimization of Structured Illumination Image Reconstruction Code into the GPU Environment

**DOI:** 10.1155/2024/8862387

**Published:** 2024-02-28

**Authors:** Kwangsung Oh, Piero R. Bianco

**Affiliations:** ^1^Department of Computer Science, College of Information Science & Technology, University of Nebraska Omaha, Omaha, NE 68182, USA; ^2^Department of Pharmaceutical Sciences, College of Pharmacy, University of Nebraska Medical Center, Omaha, NE 68198-6025, USA

## Abstract

Superresolution, structured illumination microscopy (SIM) is an ideal modality for imaging live cells due to its relatively high speed and low photon-induced damage to the cells. The rate-limiting step in observing a superresolution image in SIM is often the reconstruction speed of the algorithm used to form a single image from as many as nine raw images. Reconstruction algorithms impose a significant computing burden due to an intricate workflow and a large number of often complex calculations to produce the final image. Further adding to the computing burden is that the code, even within the MATLAB environment, can be inefficiently written by microscopists who are noncomputer science researchers. In addition, they do not take into consideration the processing power of the graphics processing unit (GPU) of the computer. To address these issues, we present simple but efficient approaches to first revise MATLAB code, followed by conversion to GPU-optimized code. When combined with cost-effective, high-performance GPU-enabled computers, a 4- to 500-fold improvement in algorithm execution speed is observed as shown for the image denoising Hessian-SIM algorithm. Importantly, the improved algorithm produces images identical in quality to the original.

## 1. Introduction

Superresolution, structured illumination microscopy (SIM) is an ideal modality for imaging live cells due to its relatively high speed and low photon-induced damage to the cells in comparison to other superresolution fluorescence microscopy techniques [1, 2]. Structured illumination microscopy and its variants thereof are based on the original wide-field design of Gustafsson [3]. SIM consists of two generic components: (i) sample illumination by a sinusoidal pattern and (ii) computational reconstruction of a superresolution image [4]. Over the years, intensive research has focused on improving the hardware, the means of sample illumination, the algorithms to reconstruct images, and approaches to increase algorithm reconstruction speed [5–9]. The overarching goal of these combined efforts is to produce an imaging modality that produces superresolution images in real-time with minimal artifacts [1, 10–13].

Often, the rate-limiting step in observing a superresolution image in SIM is the reconstruction speed of the algorithm required to form a single image from as many as nine raw images [14, 15]. This follows because the most widely used approaches perform a Fourier transform of the captured images, then perform calculations in Fourier space, and once this is done, an inverse Fourier transform is done to produce the superresolution image. These reconstruction algorithms impose a significant computing burden due to a complex workflow and a large number of calculations to produce the final image [16, 17]. This requires several seconds (10-300 per image) which nullifies real-time imaging [7, 18]. In addition, image reconstruction calculations must be performed with great care as artifacts can be introduced into the final images, and this is further complicated by the motion of the cell or organelles during imaging [10, 16, 17, 19–21].

Until recently, most image reconstruction algorithms were executed on computer central processing units (CPUs), where instructions are executed serially. In contrast, the execution of instructions within the graphics processing unit (GPU) environment is done in a parallel fashion and is 10-100-fold faster than the CPU [22–24]. Thus, and due to the heavy computing burden, it makes sense to reconstruct superresolution images in the GPU environment. This was first demonstrated, albeit in a complex fashion, using three cameras and multiple computers, by Markwirth et al. [25]. More recently, an improved algorithm that used a simplified workflow called Joint Space and Frequency Reconstruction SIM (JSFR-SIM) was developed [26]. While this algorithm is only 2-fold faster than the widely used Wiener SIM, the conversion of code to the GPU environment resulted in a 77-fold improvement in execution speed. However, the CPU-GPU code conversion is not straightforward, and in addition, the vast majority of SIM image reconstruction code is not written by computer scientists.

Although our approach to producing “GPU-enhanced code” was initially implemented with JSFR-SIM [26], we thought it pertinent to present details of how this can be done. We describe two different GPU-enhanced desktop computers to facilitate high-speed code execution for SIM or other imaging modalities. To show how the conversion can be implemented, we selected the computational-intense Hessian-SIM as the test code and describe how to enhance algorithm processing speed within the framework of MATLAB [18]. MATLAB is a popular programming language and computing environment for many microscopy researchers as it offers an easy way to write, test, and run image-processing algorithms without background knowledge in computer science. However, the resulting algorithms can suffer from poor performance due to inefficiently written code designed to be executed in the CPU environment only, as we will demonstrate.

Image denoising, which searches for a clean from a noisy image, is one of the most important branches of image processing [27, 28]. The Hessian-SIM denoising algorithm was used to remove the fluctuation noise caused by variance [18]. While the algorithm provides excellent output image quality, the original MATLAB code performs poorly. For example, it took 133 seconds to complete the processing of a single 128 × 256 × 180 stack of 180 images (provided as raw data with the Hessian-SIM algorithm tested herein). This test was done using one of the graphic processing unit- (GPU-) enabled machines used in our experiments (described in the Materials and Methods). For real-time imaging of live cells where the motion of cellular components is on the millisecond time scale, this image reconstruction speed is too slow. This follows because the significant motion of cellular components would have occurred before image reconstruction using this approach would be complete, and thus, attempts to visualize events would be futile.

A careful examination of the code revealed that it was inefficiently written by noncomputer science researchers and without consideration of the GPU. Consequently, in this paper, we introduce simple but efficient approaches to first revise MATLAB code, followed by conversion to GPU-optimized code. These approaches used for superresolution SIM can easily be applied to other image-processing codes and could also be applied in different high-level languages such as Python and R. Note that this work targets researchers who do not have programming background knowledge and use MATLAB due to its simplicity. In addition, this paper mainly focuses on how implementations (code) of image-processing algorithms can be improved for better performance but not how the algorithms logically work. Thus, researchers can try to adapt our code-optimizing approaches to their code when they observe the performance bottlenecks incurred due to implementations but not algorithms. The combination of code improvement, conversion to the GPU environment, and use of a GPU-enabled computer result in a 4- to 500-fold improvement in algorithm execution speed, and the resulting image quality is identical to or better than that produced by the original algorithm.

## 2. Results and Discussion

### 2.1. Identifying Algorithm Performance Bottlenecks

One of the most important steps to improve the performance of image-processing algorithms is to locate the code or functions that dominate the overall execution time required to complete algorithms. If these lines of code are inefficiently written, they constitute potentially significant bottlenecks.

As MATLAB is frequently used in algorithm writing for microscopy, the easiest way to find the performance bottlenecks is to use the MATLAB Profiler. This enables researchers to profile code interactively. The Profiler measures the time to run code and lists the most time-consuming code and functions. The users can start the Profiler at the beginning of their algorithms and end it at the next line of the last lines of their code.

**Figure 1:**
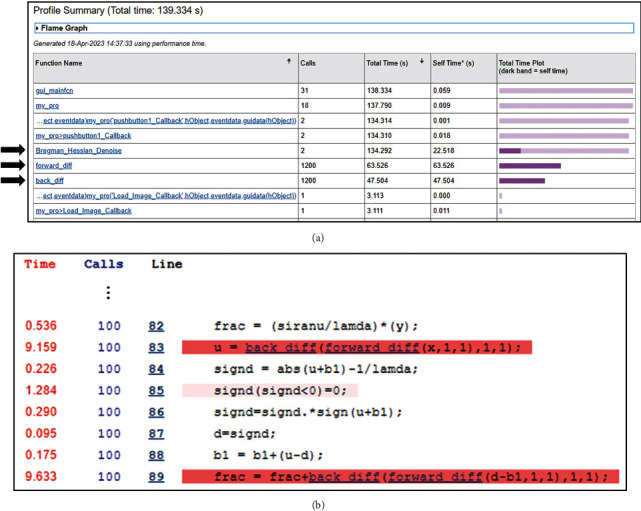
Analysis of potential bottlenecks in the original Hessian-SIM code. (a) The MATLAB Profiler generates a summary for users to enable evaluation of the potential bottlenecks in the code. (b) A snippet of the detailed profile for the Bregman_Hessian_Denoise () function showing which lines of code take a lot of time. There are similar lines of similar code for another five tasks. Red lines indicate code lines that spend more time than other lines.

In our test example, we put the “Profile on” command before the Hessian-SIM denoising algorithm initiates and put “Profile viewer” after the algorithm is complete. Once profiling is done, i.e., the “Profile viewer” command is executed, the profiling window will show up (Figure 1).

The results show that the *three functions*Bregman_Hessian_Denoise()(in the original code, Bregman_Hessian_Denoise is used as a script but not a function. We refer it as a function in the following sections for simplicity. We will explain the differences between scripts and functions in Section 2.7), forward_diff(), and back_diff() account for approximately 96 percent of the overall execution time, or 134 of the 139 seconds (Figure 1(a), black arrows). Note that the execution time will increase for profiling. Based on the report, we investigated these three functions to ascertain how the performance bottleneck occurred. We found that the Bregman_Hessian_Denoise() function includes a loop iterating 100 times. In the loop, we found that the loop contains *six independent denoising tasks* that compute values of six directions in the three dimensions (x, y, z), i.e., xx, xy, xz, yy, yz, and zz, using the same matrix **X** as an input. To see which lines of code incur performance bottlenecks, a user can click a function in the report summary (Figure 1(a)) for a detailed profile, e.g., the execution time of each line of code in the function.  Figure 1(b) shows a detailed profile of one of the tasks to update values for the xx dimension in the loop.

In addition, calling forward_diff() and back_diff() in a loop takes a lot of time. In each task, the forward_diff() and back_diff() functions are called four times (two for each), i.e., 24 times in each iteration and 2400 function calls in 100 iterations. Further, our analysis of the forward_diff() and back_diff() functions reveals that they were inefficiently written as we will discuss below. In addition, we found that a single line of the code (line 85 in Figure 1(b)) also takes more than a second, which incurs another performance degradation. These observations indicate that these functions are obvious performance bottlenecks, and if efficiently revised, performance should improve. Thus, we mainly focused on revising these functions to improve performance.

While the Profiler provides comprehensive profiling information, users need to wait until the execution is complete to obtain profiling information. To determine the elapsed time of code and function at runtime, the users can use the *tic* and *toc* functions of MATLAB. The tic() function records the current time when it is called, and the toc() function uses the recoded value by the tic function to calculate the elapsed time. Thus, the tic() function can be placed at the start of the code, and the toc function can be placed at the end of the code and functions. Once the toc() function is called, the MATLAB runtime shows the elapsed time in the Command Window. By using the Profiler and the tic-toc functions, users can measure code execution time and identify code and functions that incur performance bottlenecks.

### 2.2. Monitoring Hardware Limitations

In addition to imperfections in the code, performance bottlenecks may occur due to limited hardware resources, e.g., CPUs, GPUs, and memory. If these resources are rate-limiting, refactoring (optimizing) code may not improve image-processing performance. For example, a performance bottleneck from a GPU with a small number of cores and a small size of video memory (VRAM) may be encountered, and this bottleneck cannot be overcome by utilizing GPU-optimized code. In this case, upgrading one or more computer components will be required to achieve the desired performance improvement.

**Figure 2:**
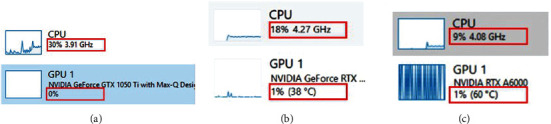
Hardware utilization monitoring is easily performed using Task Manager. A screen capture was performed during the execution of the original Hessian-SIM denoising algorithm on the three test computers used in this study. (a) Baseline machine. (b) Intel machine. (c) AMD machine. Red boxes highlight the CPU and GPU utilization by each computer during algorithm execution.

The Task Manager in Windows is a straightforward way to assess if there is a hardware bottleneck. In addition, this Windows-based utility can also reveal which components are being utilized during algorithm execution. To demonstrate this, the Task Manager was activated, and the original Hessian-SIM code was executed on three machines that we used in this work. The results show that the vanilla code of the algorithm utilizes the CPU inefficiently as utilization levels were less than 30, 18, and 9%, respectively, in our machines. In addition, the GPU was not taken advantage of at all, i.e., 0% GPU utilization (Figure 2). Note the low GPU utilization is for other processes, e.g., video output (display), and not for the Hessian-SIM algorithm execution. Based on such low resource utilization, we predict that optimizing code to take advantage of available hardware on either a standard computer or a high-performance machine would improve performance by increasing CPU and GPU utilization.

### 2.3. Memory Access and Built-In MATLAB Functions

To process images, the input images must be loaded into the registers (CPU's internal memory) from the main memory. While the CPU can access the registers and the main memory, it cannot directly access secondary storage (either HDD or SSD) that stores image data initially. Once image data is loaded into the main memory from a second storage by an operating system, the CPU can access and load it into the registers to process. Since the main memory is not part of the CPU, accessing it is much slower than accessing the registers based on memory locality. Thus, frequent accessing of the main memory such as creating matrixes and transferring data among matrixes can easily incur performance bottlenecks as the CPU cannot make progress until memory access operations are done. Thus, it is important to reduce (minimize) memory access to improve overall performance. It is also important to consider memory access patterns to maximize performance, i.e., cache memory (Supplementary Material (available here)). The same is applied to GPU. To help researchers implement their algorithms easily, MATLAB provides diverse built-in functions. These functions are highly optimized in terms of performance with consideration of diverse code optimization techniques, algorithms, and memory access. Using built-in functions will likely result in better performance compared to customized functions that could be written inefficiently.

**Figure 3:**
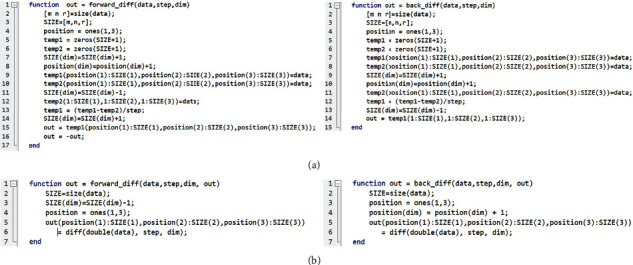
The impact of code revision. (a) Complex, unedited code. (b) Simplified and improved code. Revised code of forward_diff() and back_diff() functions that use the built-in and avoid creating memory (matrixes) in each function call. We use the built-in function, diff(), that calculates the differences between adjacent elements, and the differences are stored in a preallocated matrix (out). The matrix is passed as a parameter to the revised function to avoid creating temporary matrixes in each function call, i.e., reduced slow main memory operations. The variable, position, is created and used to indicate where to store the output of diff(), in the preallocated matrix, i.e., out. For example, in the code of the right of (b), the output location is adjusted by increasing one of the dimensions based on the third parameter (dim) by 1. This variable could be removed in the code on the left of (b) by updating the last line of code to out(1:SIZE(1), 1:SIZE(2), 1:SIZE(3). We kept using the variable to be consistent within both codes of (b). Note that the diff () function may create matrixes internally or use preallocated matrixes, which are hidden from users. We assume that the overhead would be negligible due to MATLAB's highly optimized built-in functions.

We started revising the two functions, i.e., forward_diff() and back_diff(), as they spend most of the execution time (Figure 1). We found that these functions calculate differences between adjacent elements in the matrix. To achieve this, these functions create two additional matrixes and copy the original matrix data into both newly created matrixes that are used to get differences by subtracting them (Figure 3(a)). Even though this implementation may be straightforward, it requires significant memory access for creating matrixes, copying original matrix values, and subtracting two matrixes for each function call. This significantly degrades performance. Furthermore, these functions are called 2400 times (24 × 100) in the loop with 100 iterations, which further inflates execution time.

To remedy this performance overhead, we first searched MATLAB for built-in functions to implement the same functionality. We found the diff(**X**, **n**, **dim**) function that calculates the differences between adjacent elements in a matrix. However, this function changes the dimension (**dim**) of the input matrix (**X**) acted on by diff that is reduced in size by **n** in the output. For example, for the input image X (128 × 256 × 180), calling diff(**X**, 1, 1) results in 127 × 256 × 180 as an output matrix. Such different dimension matrix requires additional work, which could be burdensome work for researchers. It is conceivable that this is the reason why the authors of the original Hessian-SIM code implemented these functions by themselves instead of using the diff() function.

We revised forward_diff() and back_diff(), to use the diff() function and preallocated matrixes (memory) for storing the results (differences), which allows us to efficiently compute the differences of adjacent elements and avoid unnecessary memory access (Figures 3(a) and 3(b)). By visual inspection, one can easily see the difference between the original and improved code. The revised functions (Figure 3(b)) take the additional parameter ( out) that will store the output of the functions. This avoids creating temporary variables in the functions by using the same memory for input and output, i.e., *in-place memory optimization*. This technique is beneficial when the input data does not need to be preserved. While we do not use the value of out as input, passing and using out in the functions improve performance by avoiding memory allocation during algorithm execution.

We observed that a single line of code requires more time than other lines, i.e., line 85: signd(signd <0) =0 (Figure 1(b)). This code converts all negative values in a matrix ( signd) to 0 using logical indexing. Since this code is executed 6 times per iteration (600 times in total), this code incurs performance overhead. To reduce the overhead, we searched for MATLAB built-in functions that can convert negative values to 0. We found the max(A, B) function that returns an array with the largest elements taken from A or B. For example, X = max(X, 0) outputs the matrix (*X*) in which all negative values are converted to 0 by comparing all the elements in the *X* to 0. Such simple code revisions can improve performance by utilizing MATLAB built-in functions as we will show in the evaluation section below.

### 2.4. Inline Code

**Figure 4:**
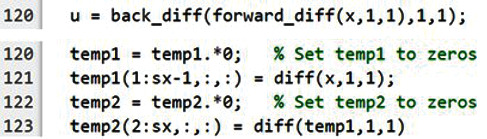
Inline code. The calling function requires additional memory access to keep machine states in memory, called a stack. Thus, frequent function calls will result in frequent memory access, which results in performance degradation. The forward_diff() and backward_diff() functions are called 24 times in each iteration in the loop, which incurs significant performance overhead. This figure shows that the function body can be directly used instead of calling functions, i.e., inline code. Note that the dimension of the input acted on by the diff () function is reduced in size by *n* in the output. To preserve the matrix size and avoid creating temporary variables, we use two preallocated matrixes (temp1 and temp2) and store the reduced matrix (output of diff()) using array indexing. We set the preallocated matrixes (temp1 and temp2) to zeros, i.e., temp1=temp1.∗0, before calling the diff() function as the original Hessian-SIM code fills zeros to the reduced dimension. Note that we use element-wise multiplication ( .^∗^) to set a matrix to zeros as we found that it offers better performance than other known approaches, e.g., $temp1\left(:\right)=0$.

While using built-in functions to improve performance, they are still used in forward_diff() and back_diff() functions multiple times (2400 times in total) in the loop. Since every function call requires memory access for stack operations to keep states of algorithms, frequent function calls would incur additional performance overhead. To address this issue, we revised the Bregman_Hessian_Denoise() function to call diff() and max() directly in the loop instead of calling forward_diff() and back_diff(), that is, using inline code, which is a technique that replaces a function call with the contents (body) of that function (Figure 4). In this manner, unnecessary memory access for function calls is reduced, further improving performance.

### 2.5. Duplicated Operations

One of the most common mistakes observed in inefficiently written code is conducting redundant operations in a loop (iteration). If the value is known to be constant and used in loops repeatedly, it should be computed only once to avoid redundant computation. This follows because redundant operations require more execution time, thereby degrading performance.

From the six tasks in the loop of the Bregman_Hessian_Denoise() function mentioned previously, we found that the same function with the same input and parameters was called multiple times in a loop, e.g., diff (X, 1, 1) three times in each iteration. Since the results are the same for each function call, we keep the result of the function call in the preallocated memory (matrix) at the beginning of the iteration, e.g., temp_m = diff (X, 1, 1), and use temp_m when the matrix is needed, thereby eliminating redundant operations.

The Bregman_Hessian_Denoise() function initializes the FFT of the difference operator of which values are determined based on input image size. That is, if the input image size is the same, these lines of code result in the same value, i.e., constant values based on image size. To avoid duplicated calculations for the same image size, we store the results (constant values) in persistent (secondary) storage, such as the HDD or SSD. Note that we choose the filename for the values based on image size to avoid any overwriting of files, e.g., 128-256-180.mat. Once these values are placed in storage, they can be read from storage from the next execution instead of calculating them. This approach may provide marginal benefits only when computing constant values takes more time than reading them from storage. However, such a small performance improvement would be highly appreciated for the latency-strict image progressing algorithms targeting less than a few hundred milliseconds. We will show the benefit of this approach in the GPU-enabled revised code in Section 2.10.

### 2.6. Floating-Point Number Precision

Any real number that has an infinite number of digits such as 1/3, the square root of 2, and PI cannot be represented completely since there are only a finite number of bits for storing them. Computers use the floating-point representation (IEEE754) citation [29], either 32-bit (single) or 64-bit (double), to represent real numbers by finding the closest numbers of real numbers, which results in precision issues. For example, the real number 0.3 is represented as follows.

- Single precision: 0.300000011920928955078125
- Double precision: 0.299999999999999988897769753748434595763683319091796875

MATLAB uses double-precision floating-point numbers by default. For example, X = zeros(10, 10) generates a 10 × 10 double-precision matrix *X*. In general, computing single-precision floating numbers should be considerably faster because it uses half memory compared to double, which requires fewer floating-point operations. Note that the performance of calculating floating numbers could vary based on hardware architectures, e.g., CPU and GPU, that determine how they are computed. For latency-critical algorithms where single precision is sufficient, MATLAB allows users to use single-precision floating numbers. For example, users can use a simple conversion function, e.g., X = single(X), or put the argument “single” when creating a matrix, e.g., *X* = zeros(10, 10, ‘single').

In the Hessian-SIM denoising code, we found that single-precision matrixes and double-precision matrixes are created and used together for computation, e.g., the sum of a single-precision matrix and a double-precision matrix. In this case, the result is always a single-precision matrix, which makes double-precision useless. In addition, this requires MATLAB to convert double-precision numbers to single-precision numbers at run-time, which incurs additional overhead. Thus, we revised the code to use single precision for all matrixes to improve performance. This approach would result in outputs that are not binary identical compared to the original code due to precision issues to represent real numbers. Based on the Hessian-SIM code that uses both single and double precision interchangeably, we suggest that such differences are acceptable. Note that there are similar precision issues between the CPU and the GPU due to different hardware architectures and algorithms for computing floating-point numbers, which is unavoidable.

For creating a single-precision matrix, using the zeros() function with the “single” parameter, e.g., X = zeros(1000, 1000, ‘single'), provides much better performance than using the single() function, e.g., *X* = single(zeros(1000, 1000)). For example, X = zeros(1000, 1000, ‘single') takes 0.071 milliseconds while X = single(zeros(1000,1000)) tasks 1.267 milliseconds in one of the machines that we used for evaluating our approaches. This is because the latter code creates a double-precision matrix first and converts it into a single-precision matrix while the former code creates a single-precision matrix from the first.

### 2.7. Code Structure: Scripts vs. Functions

For the sets of MATLAB code that are repeatedly used, users can create either scripts or functions. While both allow users to reuse the same code, they work differently, which affects performance. The scripts are ordinary MATLAB files (∗.m) containing codes. To execute scripts, users can type the script filename (without extension) in the Command Window, double-click the script file, or put the filename in the code. Scripts work like functions, but they do not take parameters and return values. Functions contain sets of codes like scripts but are declared by MATLAB keywords, i.e., start with “ function” and end with “ end.” Unlike scripts, functions take parameters and return values, which gives more flexibility and extensibility to users.

In terms of performance, functions generally provide better performance than scripts because of the reduced search spaces for lookup variables. Each variable has its lifetime and scope based on where they are created. Variable lifetime indicates whether a variable is loaded into memory to be used by the CPU and variable scope determines the code region where the variable is visible (accessible). If a variable is created in a script, it is a “*global*” variable that starts its life when the scripts are executed in the base workspace and is visible throughout the scripts' execution. Note that the base workspace size increases when new scripts are executed. When a global variable needs to be accessed, it needs to be searched from the large base workspace, which yields performance overhead due to the large search time. On the other hand, a variable created in functions is a “*local*” variable that starts its life when the functions are called in a function workspace (separate from the base workspace) and is visible only within that function, which has a narrowed search space, which offers better performance compared to searching global variables.

**Figure 5:**
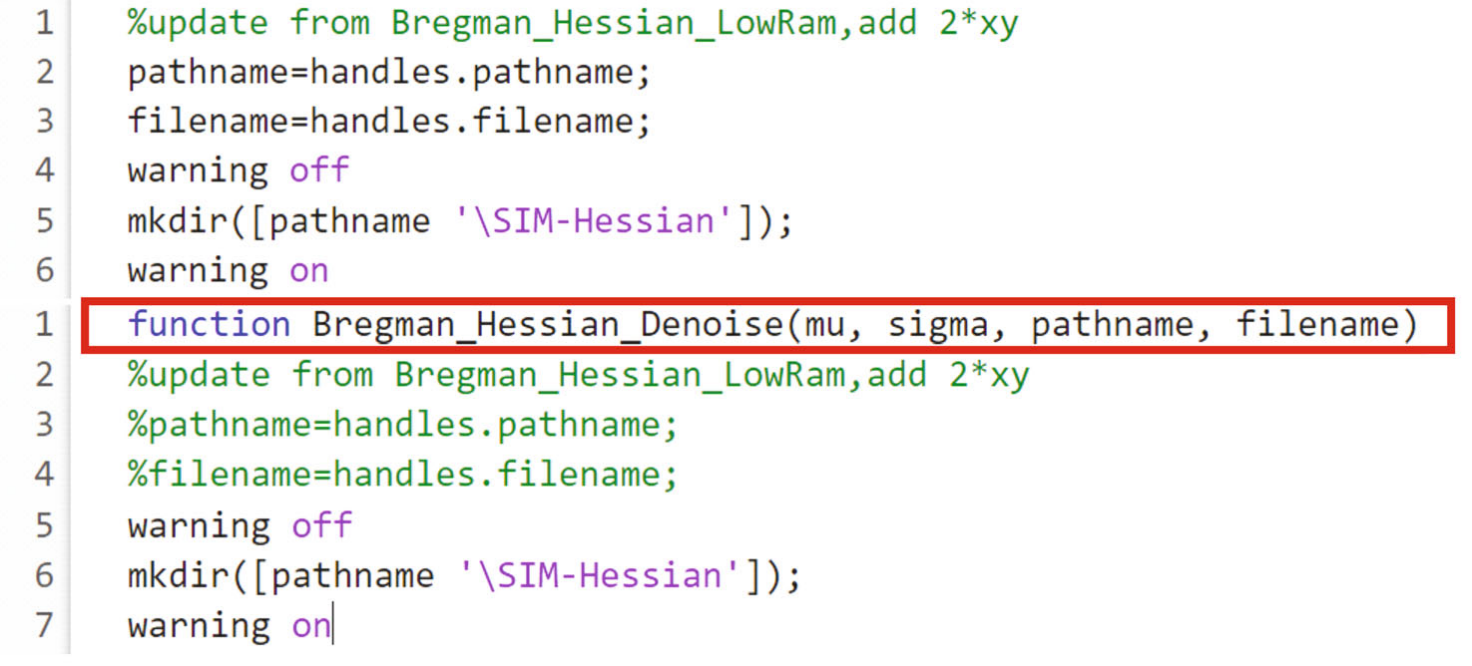
Converting script file into a function to reduce the workspace to search variables. Note that the figure shows the code snippet. The parameters of the Bregman_Hessian_Denoise () function, i.e., mu, sigma, pathname, and filename, are used later in the function.

Code analysis shows that the Bregman_Hessian_Denoise is executed as a script instead of a function, which incurs a performance overhead due to a larger search space. To reduce the search time, we converted the script to a function using MATLAB keywords, i.e., “ function” and “ end” for the Bregman_Hessian_Denoise() function.  Figure 5 shows the simple code revision to create a function to exploit a reduced-size workspace for better performance.

Note that the Bregman_Hessian_Denoise function (script) is called only once, and thus, the overhead from the function call is negligible. This approach offers marginal performance improvement compared to vanilla code as the dominant bottlenecks come from the other sets of code. This approach is highly desirable for latency-sensitive image-processing algorithms that should be executed in several hundreds of milliseconds. We will show the benefit of this approach in GPU-enabled revision code in Section 2.10.

### 2.8. Concurrency: Exploiting Multiple CPU Cores

Most recent CPUs offer multiple processing units, i.e., CPU cores. For example, there are 12, 24, and 64 CPU cores in the machines that we used in this work. Each CPU core can process different tasks independently, i.e., multitasking. Thus, multiple CPU cores can be used for image-processing algorithms to improve performance by processing different tasks in parallel, i.e., concurrency. To achieve concurrency in image-processing algorithms, researchers must know how to write efficient code with consideration of multicores, which can make the code more complex.

**Figure 6:**
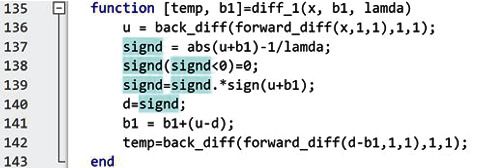
A function to exploit parallel computing with multiple CPU cores. MATLAB supports parallel computing using multiple CPU cores. To utilize multicores, users need to create functions that are to be executed in different CPU cores concurrently and independently. The figure shows a function of xx direction task among independent six tasks. Note that the other five functions have similar patterns of code. The body of functions is placed in a loop together. We split the code into six tasks and use function and end keywords to create functions for each task.

MATLAB offers an add-on product called the Parallel Computing Toolbox (PCT) that allows users to easily utilize multicores for parallel computing by making a parallel pool. As mentioned earlier, there are six tasks in the loop of the Bregman_Hessian_Denoise() function, and they can be executed independently. Thus, six tasks can be executed in separate six CPU cores in parallel. To run these six tasks in parallel, we use parfool(resources, poolsize) function and parfeval(fcn, numout, X1,…, Xm) function provided by the PCT. The parfool() function starts a parallel pool of workers (CPU cores), and the parfeval() function schedules the function fcn to be executed asynchronously. We revised the code to create six separate functions for six tasks to be executed in each core in parallel.  Figure 6 shows one of the six functions for a xx direction task while the other functions have similar code.

Users may want to use the parfor() function to run each iteration in parallel. For example, for the loop with two iterations, the first and second iterations can be executed in parallel in different CPU cores using the parfor() function if there is no dependency between them. However, in the loop in the Bregman_Hessian_Denoise() function, each iteration depends on the previous iterations, e.g., the second iteration uses the output from the first iteration as an input. Thus, we used the parfeval()function to run six tasks in parallel in each iteration.

For a parallel pool, MATLAB allows users to set the parallel environment, either process-based (default) or thread-based environments, which offers different advantages. The process is a program in execution that uses independent resources including memory. Thread is a lightweight process (segment of a process) that shares process resources. That is, multiple threads can be executed in a single process. In both environments, tasks can be executed in separate CPU cores. We first set and use the process-based environment where each task is executed in different processes. We found some performance improvement compared to vanilla code as six tasks can be executed in parallel in different CPU cores. Then, we set and use the thread-based environment where each task is executed in different threads that share process resources. We observed better performance from the thread-based environment, e.g., 63 seconds from the thread-based environment and 85 sec in the process-based environment in one of our machines. We predict that this is because of inefficient memory usage in the process-based environment. That is, each process of each task creates matrixes (memory) independently, which requires a large memory creation and data transfer among processes to complete algorithms. Researchers should use the thread-based parallel pool if the algorithms can be completed within a single process to share memory without communication with other processes (or computers).

Executing tasks in parallel with a parallel pool, unfortunately, may not result in performance improvement if each task is too small (completed quickly). This is because parallel processing for concurrency requires additional overhead to manage multiple workers (either process or thread) in a thread pool such as splitting tasks, assigning tasks to cores, and combining the results. To get benefits from parallel processing with multiple CPU cores, each task must be large enough. Otherwise, the overhead of parallel processing would increase and thereby degrade performance. That is, the overhead for using parallel processing is greater than the performance gains, which would inflate the overall execution time. Note that there is a one-time cost to make a parallel pool active in MATLAB. We will discuss massive parallelism using GPU in the following sections.

### 2.9. Implementation of the GPU

In many image-processing algorithms, each image pixel can be processed independently of each other. While the CPU provides parallel computing with multiple complex and powerful cores, e.g., 64 CPU cores, it is designed for general-purpose processing in sequential order. Since processing a pixel is a very simple and short task, using CPU cores for calculating each pixel would incur a significant performance penalty due to the overhead for parallel computing for small tasks as we discussed in the preceding.

In contrast, the GPU is designed for massive parallelization with numerous single-purpose (graphics processing) cores, e.g., 10,752 GPU cores in the Nvidia RTX 3090. Each GPU core can compute each pixel independently in parallel, which works well for most image-processing algorithms. Thus, it is highly desirable to exploit the GPU for image-processing algorithms to improve performance. The MATLAB add-on, Parallel Computing Toolbox (PCT), also allows researchers to easily exploit the GPUs.

**Figure 7:**
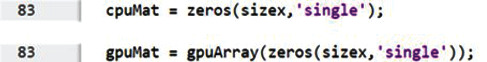
Create a matrix in CPU memory and GPU memory. The CPU can access CPU memory only, and the GPU can access GPU memory only. Data (images) must be loaded into corresponding memory to be processed by either CPU or GPU. MATLAB also supports creating a matrix (double precision) in GPU directly by passing a parameter, e.g., zeros (sizex, ‘gpuArray'). However, images cannot be directly loaded into GPU, which requires that images need to be loaded into CPU memory first and copied to GPU memory.

With PCT, utilizing the GPU in MATLAB is straightforward. Users need to simply wrap each matrix with the gpuArray() function which copies the data (matrix) stored in CPU memory into GPU memory automatically. Since the CPU and the GPU have their memory independently, data such as matrix in CPU memory must be loaded to GPU memory to be computed by the GPU. The matrix can be stored in GPU memory using the gpuArray() function (Figure 7).

Most MATLAB built-in functions that accept matrixes as inputs check the input data type to see if data is stored in either CPU or GPU memory. Based on data locations, they automatically exploit either the CPU or the GPU. For example, if the variable gpuMat in Figure 7 is passed to the diff () function, MATLAB uses the GPU to compute the differences of each adjacent pixel in parallel, which improves performance significantly.

While using MATLAB's built-in functions including the gpuArray()function to easily exploit the GPU to produce a performance improvement, the performance gain could be limited due to inefficiently written code. Therefore, to maximize the benefits of the GPU, all approaches that we introduced in previous sections must also be applied to GPU-enabled code as well.

Since the architectures of the CPU and the GPU are different, it is important to understand that GPU operations execute asynchronously to the CPU, which means that the CPU and the GPU can run simultaneously for different codes. After the CPU offloads computing tasks to the GPU, the CPU executes the next line of code instead of waiting for the results from the GPU, i.e., asynchronous execution. If the CPU continuously offloads the tasks to the GPU, the tasks are queued and executed sequentially in the GPU. When the CPU needs to get the results from the GPU, the CPU needs to call the gather() function. The function will wait until all offloaded tasks to the GPU are done and copy the result data in GPU memory into CPU memory.

To measure the execution time for GPU operations, users may want to use the tic-toc functions. In this case, the measured elapsed time may not include the actual GPU operations as the CPU keeps executing the code asynchronously. Thus, tic-toc functions should include the functions, e.g., the gather() that makes the CPU wait for results from the GPU to measure elapsed time correctly. Users can use the gputimeit(F) function to measure the elapsed time to run the function ( F) in the GPU.

In a GPU-enabled environment, data needs to be transferred between CPU and GPU. However, data transfer between CPU and GPU memory requires a lot of time due to the limited bandwidth between these components. In addition, memory access is slower than CPU and GPU performance as we discussed. Thus, frequent data transfer between the CPU and the GPU during algorithm execution incurs performance overhead and thus must be avoided. To avoid unnecessary data transfer, all data required for image-processing algorithms in CPU memory can be copied into GPU memory a priori, i.e., preallocating. This makes all data processing done in the GPU without additional CPU memory access, which would improve performance significantly.

### 2.10. Impact of Code and Hardware Improvements on Improved Hessian-SIM Execution Time

To measure the elapsed time for executing the Hessian-SIM algorithm, we used the latest version of MATLAB (R2023a). We used the unedited code as a performance baseline for the sake of performance comparisons and then demonstrated the impact of code revision and conversion to the GPU environment on algorithm performance. We applied our approaches independently to the Hessian-SIM code to demonstrate the performance improvement from each. In addition, the impact of two cost-effective high-performance computers on performance, relative to a baseline computer, is also shown.

The three computers tested are a Dell notebook computer which we consider as the baseline machine (Intel Core i7 8750H (12-Cores), 32 GB, Nvidia GeForce GTX 1050 TI with Max-Q (4 GB VRAM). Performance is compared to the two performance machines, an Intel Core i9 12900K (24-Cores), 64 GB, Nvidia GeForce RTX 3090 (24 GB VRAM), and an AMD Ryzen Threadripper 3990X (64-Cores), 128 GB, 2 x Nvidia A6000 (total 96 GB VRAM).

**Figure 8:**
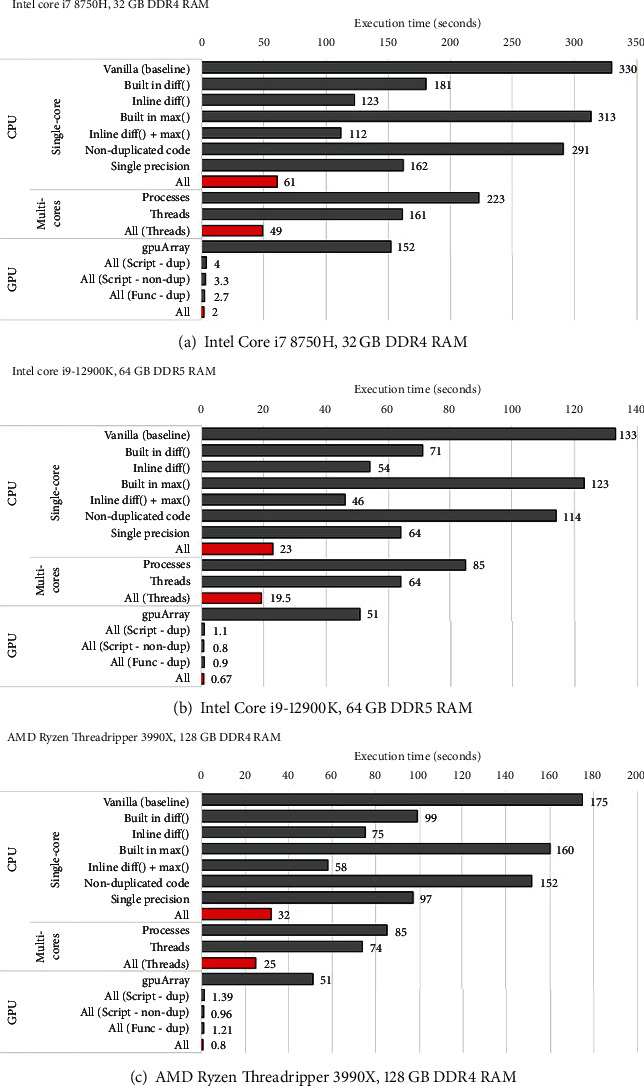
Impact of improved hardware and code on algorithm execution performance. We first measured the elapsed time of the vanilla code with a given image as a performance baseline from all the machines. Then, we applied each approach independently and measured the elapsed time to show the performance improvement of each approach. We applied all approaches with the single CPU core in the CPU-Single-core-All case. The CPU-Multicores-Process and CPU-Multicores-Threads cases show elapsed time when each task is executed in different cores without applying other approaches (red bar number 1). We applied all the approaches including multicores with which six tasks are executed in different cores, i.e., the CPU-Multicores-All case, which shows the best performance without exploiting the GPU (red bar number 2). The GPU-gpuArray case shows the elapsed time when we utilize the GPU by using gpuArray() function only without applying other approaches. This case clearly shows that performance improvement is limited even with the GPU if the code is written inefficiently. The GPU-All (Script-dup) case, the GPU-All (Script-non-dup) case, and the GPU-All (Func-dup) case show the benefits of avoiding duplicated operations and utilizing functions instead of scripts. While the performance improvement from these approaches was marginal in CPU-only code, they affect overall execution time significantly in GPU-optimized code when the execution time is less than a second. The GPU-All case shows the elapsed time with all approaches that we introduced in this work, and the best performance we can achieve (bottom red bar in each image panel).

Results show that the improvements in the code described in the preceding sections result in an approximate overall 7-fold increase in processing speed on all computers (Figure 8). The original code requires 330 sec execution time on the baseline computer, and this decreases to 61 sec using improved code on a single core of the CPU. On the Intel i9 computer, execution time decreases from 133 to 23 sec while on the AMD-based machine, the improvement is from 175 sec to 32 sec. These results indicate that code improvement, without conversion to the GPU environment, or upgrading of hardware, produces a 5- to 6-fold improvement in processing speed. Utilization of multiple cores on each machine results in a further improvement of 12, 3.5, and 7 sec, respectively, but this is only a modest, 1.3-fold improvement.

Not surprisingly, the switch from the CPU to GPU-based environments results in the largest jump in performance. It is 24.5-fold on the base computer, 29-fold on the i9 machine, and 31-fold on the AMD machine. This represents a 165-, 192-, and 218-fold overall improvement in execution speed relative to the starting Hessian-SIM code executed in the CPU environment.

In addition to code improvement, it is important to note that the hardware configuration impacts algorithm performance as well. The baseline machine requires 330 sec to process the 180-frame image stack using the original Hessian-SIM code, whereas the i9 and Ryzen Threadripper machines are 2.48- and 1.4-fold faster, respectively, requiring only 133 and 175 sec. When multiple CPU cores are used, the improvement is 7-fold for all machines, relative to the processing speed of the original code. Finally, when the improved code is converted to the GPU-optimized form, the execution speed increases 24.5-, 29-, and 31-fold for the base, i9, and Ryzen-based machines, respectively.

**Figure 9:**
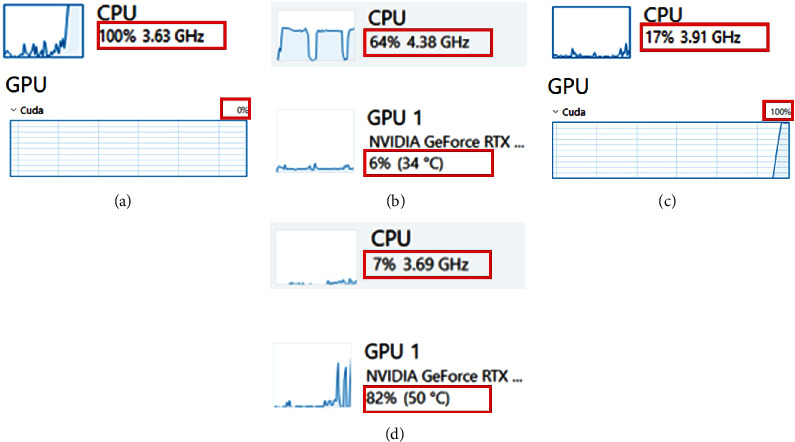
The Task Manager reveals the changes in hardware utilization by code improvement. A screen capture was performed during the execution of the improved Hessian-SIM denoising algorithm on the three test computers used in this study. (a, b) Multicores CPU utilization on the base and Intel machines, respectively. (c, d) All GPU approaches on the same computers. The red boxes highlight the relevant hardware components.

In addition to the speed improvement, the improvements in the code also demonstrate improved utilization of hardware components. To demonstrate this, the Windows Task Manager was operated during improved algorithm execution (Figure 9). First, the impact of all CPU cores was assessed, and results show that for the notebook PC, CPU utilization increased from 30 to 100%, whereas for the Intel machine, the increase was 3.6-fold from 18 to 64% (Figures 9(a) and 9(b)). When the impact of all code improvements and execution in the GPU environment was assessed, GPU utilization increased from ≤1% to 100% for the notebook to 82% for the Intel machine (Figures 9(c) and 9(d)). At the same time, CPU utilization decreased 5- and 9-fold to 17 and 7%, respectively.

In summary, code revision as described in the preceding sections improves performance (that is algorithm execution speed). If code remains in the CPU environment, this may not require hardware upgrading. However, further improvements to the code to utilize the GPU result in significant performance improvements, and this is further enhanced when the computer hardware is upgraded.

The results from the GPU also show that small performance improvements, e.g., several hundreds of milliseconds, would be highly appreciated to reduce the overall execution latency of the GPU-enabled optimized code as the GPU categories in Figure 8 indicate. For example, results from one of the cost-effective high-performance computers (Figure 8(b)) show that avoiding duplicated operations (GPU-All-Script-non-dup) reduces 0.3 sec, and using a function instead of a script (GPU-All-Func-dup) reduces 0.2 sec, which reduces 0.43 sec in total compared to the case using script and duplicated operations (GPU-All-Script-dup).

### 2.11. Impact of Improvements on the Resulting Image(s)

While we have demonstrated that our approaches improved the Hessian-SIM denoising algorithm execution speed significantly, it is critical to determine if the quality of the final image is unaffected by code changes and hardware utilization. To assess this, we compared output images generated after applying each approach using Beyond Compare [30] and Fiji [31].

**Figure 10:**
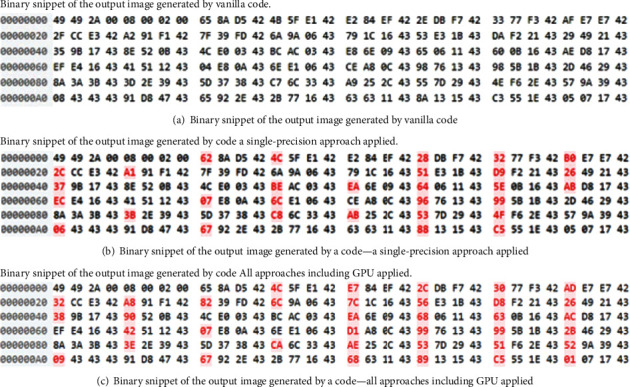
Byte differences due to the precision issues of floating-point numbers to represent real numbers. (a) Analysis of an image produced by the original code. (b) Single-precision approach image. (c) Analysis of an image produced by the improved and GPU-enhanced code. The red-colored numbers in (b) and (c) show the byte differences from the original output (baseline) image, which occurred every 4 bytes. We propose that this is acceptable based on the original vanilla code that used the single-precision and double-precision intermixed.

We confirmed that our approaches except for applying single precision for the CPU (both single core and multiple cores) yield binary identical output images (same results), confirming that performance can be improved significantly with no output changes. We used Beyond Compare 4 to compare two images byte by bytes if they are the same or not. However, using a single-precision approach and exploiting the GPU resulted in nonbinary identical images due to precision issues discussed in previous sections.  Figure 10 shows the binary differences among three images generated with (1) vanilla code, (2) single-precision approach code, and (3) GPU-All approach code. While these figures show one byte difference occurrence every four bytes, we suggest that such differences are acceptable based on the vanilla code which uses single- and double-precision numbers intermixed.

**Figure 11:**
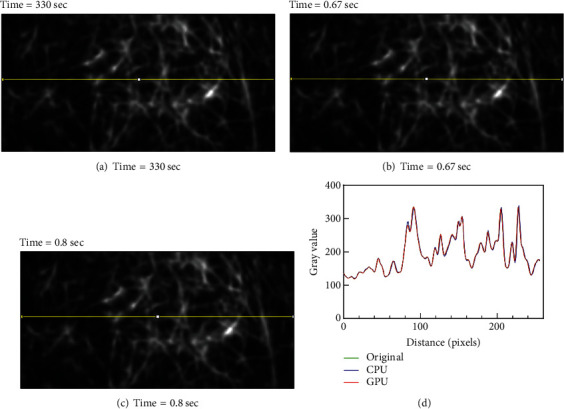
The combination of improved hardware and code produces an image stack identical to the original Hessian-SIM denoising algorithm, but at 4- to 500-fold faster rates. (a–c) Frame 50 from the image stack produced using the (a) original algorithm and (b, c) the improved algorithms, executed on superior cost-effective computers as shown in (d). Line profile analysis was performed using the Plot Profile function in Fiji [31]. The resulting data were exported into GraphPad Prism (v8.43) and displayed on the same axes for direct comparison.

To confirm that our approaches do not degrade output image quality, we compared these nonbinary identical output images to the original result. To do this, we selected frame 50 from each stack and performed line profile analysis using the Plot Profile function in Fiji (Figure 11) [31]. To try to analyze as much of the image as possible, the line profile covered the length of the image in the approximate center. Results show that the image quality produced by the improved algorithms is identical and independent of the high-performance hardware used (Figure 11(d)). Additional analysis of the test image stack was done using the SNR plugin in Fiji. This plugin evaluates the signal-to-noise ratio (SNR), peak signal-to-noise ratio (PSNR), root mean square error (RMSE), and mean absolute error (MAE). Here, the entire image stack of 180 frames from the original code was compared with improved algorithms executed on the GPU-enhanced computers. The data for each frame was then averaged to permit facile stack-to-stack comparison. The SNR is 130.32 ± 0.8; PSNR is 138.72 ± 0.9; RMSE is 5.37 × 10^−5^ ± 4.1 × 10^−6^, and MAE is 4.28 × 10^−5^ ± 3.72 × 10^−6^. Thus, even though the line profile analysis shows that the images are identical, this analysis indicates that the GPU-enhanced algorithms are superior to the original.

## 3. Conclusion

Our primary conclusion is that the performance of image-processing algorithms in structured illumination microscopy can be enhanced by improving the code, utilizing the GPU environment, and purchasing a single, cost-effective, high-performance computer.

The results show that a significant improvement can be obtained by first improving the code of the algorithm with the help of computer scientists. This will eliminate most, if not all, software bottlenecks, in the original algorithm. Then, once potential hardware bottlenecks have been identified, the code must be further improved to utilize available hardware more efficiently, taking advantage of multiple versus single cores for example as well as using cache. A significant component of hardware utilization is the use of the GPU. In the original Hessian-SIM code, the GPU was not used at all. When taken advantage of, a 165- to 218-fold overall improvement in execution speed was observed. Finally, the improved code can then be executed on a cost-efficient high-performance computer. The combination of improved code and superior hardware results in maximum performance improvement.

The approaches described herein can be generally used for performance improvement for noncomputer science specialists. We recognize that there may be further room for performance improvement utilizing MATLAB expertise as indicated in the Supplementary Information. In addition, it is conceivable that additional speed improvement may be observed when the computer contains two GPUs or when a cluster is utilized. As our AMD machine contains two Nvidia A6000 graphics cards, we are currently evaluating the potential for further speed improvements using this hardware.

While we recognize that speed is of the essence for live cell imaging, it is critical to determine whether any of the improvements in execution performance impact the quality of the resulting image. Critically, the results show that the improvements implemented herein have no detectable impact on the final image while enhancing algorithm execution speed 4- to 500-fold (Figure 11). Finally, we suggest that the high-performance computer can be used to control all microscope and camera functions with both image capture and analysis being performed on one machine. This is an efficient and cost-effective approach to providing high-speed superresolution image formation.

## 4. Materials and Methods

### 4.1. Computers

We used three different machines to show performance improvement. The first unit was a laptop designed and built by Dell (XPS 15 9570) (Intel Core i7-8750H, 32 GB of DDR 4 RAM; 1 SSD (2 TB Samsung SSD 970 EVO Plus)). Finally, the system contains one NVIDIA GeForce GTX 1050 Ti with Max-Q Design. The operating system is Windows 10. Note that this laptop is designed for energy-saving for longer battery life, which yields weaker performance than desktop computers.

The second unit was designed and built by Dell (Precision 3660). The components include an Intel W680 (Alder Lake-S PCH) motherboard; an Intel Core i9-12900K CPU, 64 GB of DDR5 RAM; and 2 SSDs (1 TB NVMe SK Hynix and 4 TB Seagate ST4000DX005). Finally, the system contains one NVIDIA RTX 3090 graphics card with 24 GB of GPU memory. To ensure sufficient power, a 750 W power supply is used. The operating system is Windows 11.

The third machine was designed by our groups and built by Digital Storm. The components include an ASUS ROG Zenith II Extreme Alpha motherboard; an AMD Ryzen Threadripper 3990X CPU, 128 GB of DDR4 RAM; and 3 SSDs (1 TB Samsung 970 EVO Plus; 2 TB Samsung 860 Pro and a 4 TB Samsung 860 Pro). Finally, the system contains two NVIDIA RTX A6000 graphics cards with 48 GB of GPU memory each. To ensure sufficient power, an 850 W power supply is used. The operating system is Windows 11.

### 4.2. Software

The MathWorks, Inc. MATLAB, version (R2023a); Fiji [31]; GraphPad Prism v. 8.43 (GraphPad Software LLC); Beyond Compare 4 [30].

## Data Availability

All MATLAB code of the presented approaches and other related materials including result images are available on GitHub https://github.com/mc2lab/GPU-enabled-Hessian-SIM. Microscopy researchers who are interested in this work can download the revised code to learn how to apply the approaches to their code to reproduce the results.

## Additional Points

*Brief Summary Statement*. Image-processing algorithms play an important role in microscopy research to generate superresolution output images. Many microscopy researchers who do not have computer science background knowledge and programming skills have used MATLAB which allows them to easily write diverse algorithms. However, they may also easily encounter a performance bottleneck (a large amount of time to complete the algorithms) due to their inefficiently written code. In this work, we introduce simple but efficient approaches that allow microscopy researchers to write efficient MATLAB code, which could improve image-processing algorithms' performance. The experimental results using one of the popular open-sourced image-processing algorithms (Hessian-SIM denoising) show significant performance improvement (up to 500-fold) by applying the presented approaches.

## Supplementary Materials

Supplementary Materials In this section, we briefly discussed how the performance can be further improved using approaches that we have not yet implemented. These improvements include (1) cache performance, (2) MEX functions, (3) porting MATLAB code into C and C++, (4) exploiting multiple GPUs, and (5) Additional performance improvement opportunities from MathWorks [32–34].

## References

[1] Heintzmann R., Huser T. (2017). Super-resolution structured illumination microscopy. *Chemical Reviews*.

[2] Hirano Y., Matsuda A., Hiraoka Y. (2015). Recent advancements in structured-illumination microscopy toward live-cell imaging. *Microscopy*.

[3] Gustafsson M. G. (2000). Surpassing the lateral resolution limit by a factor of two using structured illumination microscopy. *Journal of Microscopy*.

[4] Kner P., Chhun B. B., Griffis E. R., Winoto L., Gustafsson M. G. (2009). Super-resolution video microscopy of live cells by structured illumination. *Nature Methods*.

[5] Curd A., Cleasby A., Makowska K., York A., Shroff H., Peckham M. (2015). Construction of an instant structured illumination microscope. *Methods*.

[6] Ma Y., Wen K., Liu M. (2021). Recent advances in structured illumination microscopy. *Journal of Physics: Photonics*.

[7] Muller M., Monkemoller V., Hennig S., Hubner W., Huser T. (2016). Open-source image reconstruction of super-resolution structured illumination microscopy data in ImageJ. *Nature Communications*.

[8] Wu Y., Shroff H. (2018). Faster, sharper, and deeper: structured illumination microscopy for biological imaging. *Nature Methods*.

[9] Zhao T., Wang Z., Chen T., Lei M., Yao B., Bianco P. R. (2021). Advances in high-speed structured illumination microscopy. *Frontiers in Physics*.

[10] Fan J., Huang X., Li L., Tan S., Chen L. (2019). A protocol for structured illumination microscopy with minimal reconstruction artifacts. *Biophysics Reports*.

[11] Pospíšil J., Fliegel K., Klíma M. Analysis of image reconstruction artifacts in structured illumination microscopy.

[12] Smith C. S., Slotman J. A., Schermelleh L. (2021). Structured illumination microscopy with noise-controlled image reconstructions. *Nature Methods*.

[13] Wen G., Li S., Wang L. (2021). High-fidelity structured illumination microscopy by point-spread-function engineering. *Light: Science & Applications*.

[14] Heintzmann R., Gustafsson M. G. (2009). Subdiffraction resolution in continuous samples. *Nature Photonics*.

[15] Sahl S. J., Balzarotti F., Keller-Findeisen J. (2016). Comment on "extended-resolution structured illumination imaging of endocytic and cytoskeletal dynamics". *Science*.

[16] Chu K., McMillan P. J., Smith Z. J. (2014). Image reconstruction for structured-illumination microscopy with low signal level. *Optics Express*.

[17] Wicker K. (2013). Non-iterative determination of pattern phase in structured illumination microscopy using auto-correlations in Fourier space. *Optics Express*.

[18] Huang X., Fan J., Li L. (2018). Fast, long-term, super-resolution imaging with Hessian structured illumination microscopy. *Nature Biotechnology*.

[19] Forster R., Wicker K., Muller W., Jost A., Heintzmann R. (2016). Motion artefact detection in structured illumination microscopy for live cell imaging. *Optics Express*.

[20] Schaefer L. H., Schuster D., Schaffer J. (2004). Structured illumination microscopy: artefact analysis and reduction utilizing a parameter optimization approach. *Journal of Microscopy*.

[21] Zhou X., Lei M., Dan D. (2016). Image recombination transform algorithm for superresolution structured illumination microscopy. *Journal of Biomedical Optics*.

[22] Aydin M., Uysalli Y., Ozgonul E., Morova B., Kiraz A., Gregor I., Koberling F., Erdmann R. (2020). An LED-based super resolution GPU implemented structured illumination microscope. *Single Molecule Spectroscopy and Superresolution Imaging XIII*.

[23] Gong H., Guo W., Neil M. A. A. (2021). GPU-accelerated real-time reconstruction in Python of three-dimensional datasets from structured illumination microscopy with hexagonal patterns. *Philosophical Transactions of the Royal Society A*.

[24] Lu G., Baertsch M. A., Hickey J. W. (2022). A real-time GPU-accelerated parallelized image processor for large-scale multiplexed fluorescence microscopy data. *Frontiers in Immunology*.

[25] Markwirth A., Lachetta M., Monkemoller V. (2019). Video-rate multi-color structured illumination microscopy with simultaneous real-time reconstruction. *Nature Communications*.

[26] Zhaojun Wang T. Z., Hao H., Cai Y. (2022). High-speed image reconstruction for optically sectioned, super-resolution structured illumination microscopy. *Advanced Photonics*.

[27] Meiniel W., Olivo-Marin J. C., Angelini E. D. (2018). Denoising of microscopy images: a review of the state-of-the-art, and a new sparsity-based method. *IEEE Transactions on Image Processing*.

[28] Roels J., Vernaillen F., Kremer A. (2020). An interactive ImageJ plugin for semi-automated image denoising in electron microscopy. *Nature Communications*.

[29] What every computer scientist should know about floating-point arithmetic. https://docs.oracle.com/cd/E19957-01/806-3568/ncg_goldberg.html.

[30] Scooter Software I. Beyond Compare. https://www.scootersoftware.com.

[31] Schindelin J., Arganda-Carreras I., Frise E. (2012). Fiji: an open-source platform for biological-image analysis. *Nature Methods*.

[32] Mathworks *Call C/C++ MEX Functions from MATLAB*.

[33] Mathworks *Optimization Strategies*.

[34] Matlab *Techniques to Improve Performance*.

