# Supplementary material for: Facile Conversion and Optimization of Structured Illumination Image Reconstruction Code into the GPU Environment
Source: Int J Biomed Imaging. 2024 Feb 28;2024:8862387. doi: 10.1155/2024/8862387 (PMC10917484; doi:10.1155/2024/8862387)
Supplement: Supplementary Materials — In this section, we briefly discussed how the performance can be further improved using approaches that we have not yet implemented. These improvements include (1) cache performance, (2) MEX functions, (3) porting MATLAB code into C and C++, (4) exploiting multiple GPUs, and (5) Additional performance improvement opportunities from MathWorks [32–34]. [file 8862387.f1.docx]

**Introduction to the Supplementary Information**

In this section, we briefly discussed how the performance can be further improved using approaches we have not yet implemented. These improvements include:

1. Cache performance
2. MEX functions
3. Porting MATLAB code into C and C++
4. Exploiting multiple-GPUs
5. Additional performance improvement opportunities from MathWorks

**Supplementary Information**

**Facile conversion and optimization of structured illumination image reconstruction code into the GPU environment**

Running title – GPU code conversion

Kwangsung Oh^1^ and Piero R. Bianco^2^

1. Department of Computer Science, College of Information Science & Technology, University of Nebraska Omaha, Omaha, NE, 68182, USA; [kwangsungoh@unomaha.edu](mailto:kwangsungoh@unomaha.edu)
2. Department of Pharmaceutical Sciences, College of Pharmacy, University of Nebraska Medical Center, Omaha, NE 68198-6025, USA; [pbianco@unmc.edu](mailto:pbianco@unmc.edu)

Corresponding – both authors are corresponding authors.

**Supplementary Information**

In this section, we briefly discussed how the performance can be further improved using approaches we have not yet implemented.

**Cache performance**

The memory performance is much slower than CPUs and GPUs as we discussed in the main text. To fill such a performance gap, most CPUs and GPUs contain caches that are small but fast memory inside of them. The cache can improve performance significantly due to the two types of data locality, i.e., spatial and temporal, which refers to reuses of instructions (codes) and data (matrixes) within a small amount of time duration. Recently accessed codes and matrixes are stored in caches on CPUs and GPUs, which reduces frequent memory access significantly. While caches are managed by hardware (with no control for users), the code can be written to efficiently use caches. Since MATLAB stores matrixes columns in monotonically increasing memory address, accessing column-wise could maximize cache efficiency. For example, Supplementary Information Figure 1 confirms that processing column-wise results in a 5-fold improvement in performance for the same functionality.


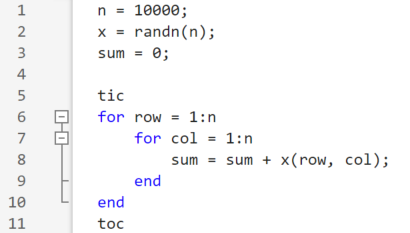

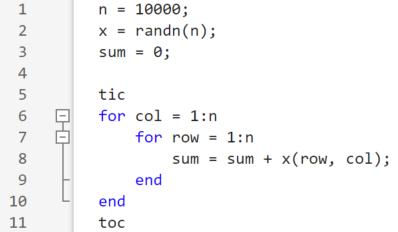

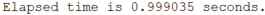

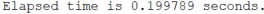


**Supplementary Information Figure 1.** **Performance can be improved by processing data column-wise in MATLAB.** Optimized code is presented on the left while unmodified code is on the right. This is because of multiple-level caches in the CPU. Since memory is much slower than the CPU, the CPU reads greater data size (cache block) from memory than needed whenever it needs to access memory and stores the data in local caches to avoid accessing nearby data next time, i.e., data locality. Since nearby columns in a matrix are likely to be stored in the blocks in caches, memory access can be reduced by accessing data column-wise. In row-wise processing, however, the next row data may not be in the cache blocks as rows are far away from each other.

**MEX functions**

While optimizing MATLAB code could improve performance significantly compared to vanilla code, there is an unavoidable performance overhead from the MATLAB runtime. This is because MATLAB is an interpreted language with which each line of code must be translated into target machine code at runtime by the MATLAB interpreter. Such performance overhead may be negligible for some image-processing algorithms which have loose performance constraints. For latency-critical algorithms, however, such interpretation overhead may hurt target performance goals, which may not be acceptable.

To avoid such overhead from MATLAB while achieving the best performance, MATLAB allows users to call C/C++ compiled (MEX) functions which avoids the overhead occurring for interpretation at runtime. A MEX function works like scripts and functions as we discussed in the previous section.

**Porting MATLAB code into C and C++**

Even with MEX functions, there would be some performance overhead from MATLAB. The programming languages having compilers, e.g., C and C++, translate the code statements into target machine code statically and create executable files that can be executed without interpretation. Thus, implementing image-processing algorithms in C and C++ would provide the best performance without having run-time overhead. In addition, using C and C++ can allow researchers to explore further optimization techniques as these languages allow users to control HW directly using diverse libraries, e.g., CUDA library for NVIDIA GPU.

However, writing MEX functions and algorithms in C/C++ could be an infeasible option for many researchers as it requires knowledge of C/C++ and HW architectures. Thus, we will not discuss it in detail. Researchers who are interested in writing C/C++ code and MEX functions may want to refer to the website (Mathworks, 2023a).

**Exploiting multiple-GPUs**

The MATLAB Parallel Computing Toolbox (PCT) allows users to utilize multiple GPUs by switching an active GPU at runtime. By assigning tasks to different GPUs, tasks can be executed independently and simultaneously on multiple GPUs. Since one of the machines we used in this work is equipped with two GPUs (2x A6000), we revised the code to execute 3 tasks in each GPU by creating two functions assigned to different GPUs. However, we could observe that running 6 tasks on two GPUs results in worse performance than using a single GPU. This is likely because the tasks are too small to be run on different GPUs, i.e., the performance overhead to switch active GPUs is greater than the benefits. Note, we connected two GPUs using NVIDIA NVLink to allow GPUs to access another GPU's memory directly. We found that MATLAB has limited support for exploiting multiple GPUs. Currently, MATLAB only supports such a high-performance direct connection for two GPUs for deep learning-related libraries but not for general built-in functions. To exploit multiple GPUs efficiently, users would need to use C/C++ code with the CUDA library.

**Additional performance improvement opportunities from MathWorks**

We found similar approaches we used in this paper are aligned with diverse Techniques introduced by MATLAB. (Matlab, 2023) Users also may want to look at the website to see other potential code optimization opportunities (Mathworks, 2023b).
